# Valuation, Categories and Attributes

**DOI:** 10.1371/journal.pone.0103002

**Published:** 2014-08-11

**Authors:** Inna Galperin, Olav Sorenson

**Affiliations:** 1 Independent scholar, Montreal, Quebec, Canada; 2 School of Management, Yale University, New Haven, Connecticut, United States of America; Utrecht University, Netherlands

## Abstract

Existing research on categories has only examined indirectly the value associated with being a member of a category relative to the value of the set of attributes that determine membership in that category. This study uses survey data to analyze consumers' preferences for the "organic” label versus for the attributes underlying that label. We found that consumers generally preferred products with the category label to those with the attributes required for the organic label but without the label. We also found that the value accorded to the organic label increased with the number of attributes that an individual associated with the category. Category membership nevertheless still had greater value than even that of the sum of the attributes associated with it.

## Introduction

Socially-constructed categories – such as occupations, organizational forms and product types – play many important roles in society. They influence the ways in which people perceive and understand the world [1] and respond to social situations [2], [3]. They affect the manner in which individuals and groups organize their activities and belongings. And they shape a wide variety of socio-economic outcomes, from the products that consumers buy [4], [5] to the people that managers hire [6] to the activities that companies pursue [7], [8].

An open question in this literature has been the relative importance of categories versus their underlying attributes in contributing to the perceived value of products, people and organizations. For example, when purchasing a computer, do consumers care about its type – perhaps a gaming machine – or do they instead mostly value a set of attributes that define that type: a fast processor, high quality video and sound processing, and a large monitor? If categories serve as cognitive shortcuts for storing and transferring information about the features (attributes) of their members [9], [10], then the value of being perceived as a member would presumably equal the combined values associated with the attributes required for membership. In other words, a buyer (perhaps subconsciously) would assign values to each of the features – the CPU, graphics processors, etc. – and the value of being considered a gaming computer would simply represent the sum of these parts (because membership in the category would imply this set of components). But categories might also have value above and beyond that of the attributes associated with them [11], [12]. Given two computers with identical components, consumers might then still pay more for the one labeled as a gaming machine.

Distinguishing between these two possibilities requires a research design that can assess the value of a category label separately from the values of the attributes that it has come to represent. To generate such independent assessments, we combined a conjoint survey instrument with an experimental design to assess the valuation of products assigned to the “organic” label relative to those that simply had the attributes associated with membership in this category. We found that respondents strongly preferred the organic label to its underlying attributes. In part, this valuation appeared to stem from associating the label with additional attractive features, ones not required for being classified as organic; respondents who associated the label with more features also placed a higher value on it. But in part, the organic label also appeared to have value in and of itself. We discuss the implications of our findings for the literatures relating classification to economic choices and outcomes.

### The Valuation of Categories

The emerging managerial and sociological literatures on categories generally assume that groups of people with similar preferences, often referred to as audiences, construct categories to sort objects, people and organizations into sets that they find meaningful, usually in the sense that they consider the members of the set to serve as functional substitutes. Thus, for example, filmgoers might classify movies into genres – action, comedy or sci-fi – based on similarities in their appeal and themes, or consumers might classify vehicles into types – minivan, sedan or sports car – based on their shapes, engine sizes and a variety of other features. Much of the recent enthusiasm about categories, however, has not been driven by wanting to understand how people develop and represent categories cognitively but by an interest in how these categories in turn shape economic outcomes. Researchers, for example, have reported relationships between the genre classifications of movies and their box office sales [5], between the previous job titles of individuals and their odds of being hired [6], and between the industry classifications of companies and the valuation of their equities [13].

Although the idea that categories would influence economic outcomes depends on the idea that categories affect the ways in which audiences value category members relative to non-members, the nature of this relationship remains relatively unexplored. We nevertheless believe that the extensive literature on why particular objects become grouped in a category hints at some possible connections between the valuations of categories relative to their underlying attributes.

### Category value  =  sum of attribute values

One common characterization of classification processes argues that an audience creates a category when a large proportion of the audience agrees that some set of producers or products shares a number of features that distinguishes them from other producers or products and attaches a label to the set [9], [10]. Thus, as noted above, an audience might perceive certain computers with fast CPUs, high-end video processors and hi-fidelity sound, large monitors and casings with lively lights and coloring as being distinct from other computing machines and label those machines as gaming computers. The category label then essentially operates as shorthand for a set of attributes or features, and category membership depends on whether – or the extent to which – an object, individual or organization has the attributes required for membership in this set [3], [10], [14].

To the extent that the category label represents a set of attributes or features, one would expect the valuation accorded to membership in the category to reflect the joint value of having all of its requisite attributes [10]. In other words, the precise set of features required for membership in a category should have a single value whether enumerated or whether specified through the shortcut of the category label. Continuing on the example above, a computer considered a member of the gaming computer category would have equivalent value to one not previously encountered (and therefore not yet classified) that had all of the features expected of such a machine (fast CPU, high-end video, etc.).

### Category value 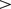 sum of attribute values

Although this equivalence between the value of a category label and of its requisite attributes seems a reasonable baseline, one might expect category labels to have value above and beyond these attributes for at least three reasons:

#### Feature creep

In many cases, categories do not have clear mappings to a particular set of attributes. People generally do not discuss categories in terms of being defined by a set of features. Instead, they attach the category label to specific cases. If discussing universities, one might give as examples Harvard, Yale, Stanford and the University of California. Inferring what features being a university requires and being able to assess whether some newly encountered organization qualifies as one depends on identifying what these accepted members of the category have in common, such as faculty, students, classes and libraries [15]–[17].

This process could lead audiences to associate categories with larger sets of features than membership in those categories actually requires. Any feature that occurs more frequently among category members than it does in the population as a whole might conceivably become associated with the category, even if only a subset of those features critically distinguish category members from non-members. Seeing that all of the examples above have campuses and dormitories, some people, for example, might come to believe that being a university requires a school to have a campus and dormitories. This feature creep might in turn inflate the value attached to membership in the category. To the extent that members of the audience place positive value on these associated features, then they may come to value the category as if it implied not simply its required attributes but also all of these additional associated ones. In other words, the value that individuals would attach to being a university might include any value that they place on having a campus or on having dormitories.

When might this feature creep become most extensive and therefore contribute most to the value associated with the category label? Interestingly, exposure to a wider range of category members probably increases the number of features that an individual associates with the category. Continuing with the university example, some accepted members of the category – but not all – would have public policy schools, medical schools, African-American studies departments and so on. One effect of being exposed to all of this variety could be to conclude that only those features that all examples have are necessary to being a university. But, to the extent that audiences and individuals view items as having grades of membership (i.e. being partial members of the category) [10], another reasonable inference would be that full membership in the category requires all of these features and that the various examples – without a public policy school or without a medical school or without an African-American studies department – have high grades of membership but are nonetheless not full members of the university category. To the extent that these associated attributes in turn have positive value, one would then expect the value of the category label to increase with exposure to examples of the category.

#### Value creep

Categories could also come to have value above and beyond that of even their associated attributes. To the extent that audiences treat categories as a gestalt, they may form their beliefs about the value of the category in a similar manner, through association as opposed to by composition from constituent parts [16]. Rather than aggregating the values of the features that they associate with a category, audiences might value the category label through inference, perhaps averaging the values that they assign to the most salient members of the category. For example, the value that an individual assigns to being a minivan might stem not from the usefulness of three rows of seats and doors that slide open and its other features but from averaging the values associated with a Honda Odyssey, a Toyota Sienna and other examples of the vehicle type. Since each of these prototypical members probably has a number of non-requisite – in the sense of not being needed to belong to the category – but desirable features, these valuations would then implicitly incorporate not just the values of features associated with the category but also those of extraneous ones.

Consistent with this notion that audiences learn the value of category membership through association rather than through composition, producers frequently attempt to communicate the value of novel products not by enumerating all of their features but rather by relating them to established categories. Manufacturers of early computer workstations, for example, first attempted to promote their products as “smart terminals” and “mainframes on a desk” [18]. Similarly, early satellite radio providers attempted to draw analogies between their services and the established offerings of cable and satellite television services [12].

In contrast to feature creep, this value creep would probably not increase with exposure to the category. Although those with more experience with the category may have a larger number of prototypical members of the category available to them and therefore may form their beliefs about the value of the category label by averaging across a larger number of examples, this averaging would not lead to a higher or lower valuation unless limited exposure implied having an unrepresentative sample. It should, however, reduce the variance in the valuation associated with the category label (as the value converges on the population average value of the category members).

#### Certification

Category labels might also come to have value in excess of that accorded to their underlying attributes due to a sort of certification value. To the extent that audiences associate a category label with a set of features, they may assume that any object, individual or organization that has received the label possesses all of these features [10]. Parents, for example, might presume that a film with a PG rating has relatively little nudity or profanity. This assumption of having the requisite features could become particularly valuable in cases where membership in the category depends on a large number of features, meaning that direct examination of the features themselves would prove costly.

By contrast, audiences might discount the value of an item, even if it has all of the features required for category membership, if it has not also been assigned the category label. After all, if producers or people met the requirements for membership in a category why would they not claim membership? Imagine a movie that proclaimed itself suitable for children but that had no MPAA rating or a school that offers the same curriculum as other business schools but that does not award its graduates an MBA. Audiences may interpret information on attributes in the absence of the category label as a signal of illegitimacy, evidence that the person or producer fails to fit in the relevant category.

### Empirical strategy

To gain greater insight into the relationship between categories, attributes and their valuations, we surveyed consumers to assess their preferences for hypothetical products with and without the “organic” category label.

The danger of focusing on a single category stems from distinguishing the valuation accorded to a category label from an incomplete or incorrect list of its constituent features. Even with a simple category, audiences may associate the category label with attributes not anticipated by the researcher. To address this issue, our empirical design exploited the fact that prior psychological research has found that – even within individual and within task – people shift between more automatic and more deliberative forms of information processing as a function of their psychological state.

In particular, people in positive moods tend to rely more heavily on generalized ideas, heuristics and theories, while those in negative moods more commonly focus on the specific data [19]. Speculation about the reason behind this effect stems from the notion that people interpret their mood as being informative of some aspect of their current situation [20]. Mood then points to the most useful processing style. When all is well, people feel good, the situation seems well understood and people can save time and energy by following tested scripts. A negative mood, by contrast, signals a problematic situation, which may require deviation from the routine and therefore demands attention to the details. One might therefore expect audiences to shift from treating categories as a gestalt to evaluating them more according to their constituent components when in a negative mood. By experimentally inducing such negative affect, we used this effect to understand better the relationship between the values of the category label and of its requisite features.

### Setting: Organics

To test these ideas, we gathered data on the perceptions of Canadian consumers regarding the value of organic poultry and its constituent attributes. This setting has a number of advantages with respect to our research question. First and foremost, though popular understanding of the meaning of the category may diverge from its official meaning, the label organic has a clear legal definition in Canada when applied to poultry. We therefore had a basis for assessing the accuracy of audience beliefs about the category. Second, as a common category of consumption, consumers have generally had to consider these choices and therefore should have some understanding both of the category's meaning and the extent to which they personally value it and its attributes.

#### Background

Interest in the “organic” production of food began in the early part of the twentieth century. Around this time, a variety of factors – the invention of the Haber-Bosch process essential to producing synthetic fertilizer, the development of synthetic pesticides, and the introduction of tractors and engine-driven equipment for planting and harvesting – converged to increase the importance of economies of scale in food production. Against this backdrop, advocacy for organic farming began essentially as a social movement opposed to the increasingly industrial nature of farming and to the treatment of the production of food as more a matter of chemistry than of biology. Interestingly, early enthusiasts came primarily from the right-wing politically, conservatives hoping to preserve traditional country life and its social order [22].

Organic agriculture began to emerge from the fringes and to shift politically from the right to the left in the 1970s, when it became attached to the environmental movement. Rachel Carson's book, *Silent Spring*, published in 1962, has often been seen as helping to instigate the environmental movement. In it, she detailed many of the devastating effects of pesticides, particularly DDT, on wildlife and humans, thereby awakening concerns among the general public about the safety of the chemicals used in modern agriculture.

Demand for food made without synthetic pesticides and fertilizers has grown steadily since then. Today, worldwide sales of organic products exceeds $50 billion per year and continues to grow at a rate far above that of the food industry as a whole [23]. Organic products moreover can be found in nearly all major grocery stores in Canada and the United States. In fact, mainstream grocery stores have accounted for the majority of organic product sales in North America since 2007 [24].

#### Organic poultry

Although the organic designation has been attached to a plethora of products, the advantages of a clear category definition demanded that we focus on a single application. We chose to study the designation organic as applied to chicken. According to the Canadian Food Inspection Agency (CFIA), three attributes distinguish organic poultry from conventional poultry:

- Organic feed: At least 80% of the feed, mostly grain, used to feed organic poultry must come from organic sources.
- No animal byproducts: Although animal byproducts commonly serve as filler and protein sources in the feed of conventional poultry, none of the feed for organic poultry can contain animal byproducts.
- No antibiotics: Once a bird has been given antibiotics, it no longer qualifies as being organic.

### Sample

To limit the potential influence of the context on our results, we gathered information from individuals engaged in a single activity: grocery shopping. This activity has the added advantage of being a context in which people must consider food categories. Three research assistants randomly intercepted individuals at five different locations – outside four grocery stores and outside one natural foods cooperative – in the greater Toronto area.

#### Ethics statement

Prior to participating in the survey, the interviewers explained the general purpose of the study and obtained both verbal and written consent, in the form of a signature on a consent form, from the subjects. The research assistants then only surveyed those who had provided written consent. Following completion of the survey, respondents received contact information for one of the primary investigators, so that they could follow up with questions or to request their removal from the study. The Social Sciences, Humanities and Education Research Ethics Board (REB) of the University of Toronto approved both the survey instrument and the consent protocol.

#### Respondents

Those who agreed to participate first had to pass several screening questions: They had to be residents of Canada, over the age of 18, who had previously purchased chicken in a grocery store. As compensation for their time, participants received five dollars in cash. A total of 576 individuals qualified on these questions and completed the survey. We excluded five surveys from the analysis—one because the respondent claimed complete indifference across all choices and four because the interviewers failed to record the responses correctly.

We conducted one-quarter of the surveys outside The Big Carrot, a local cooperative that specializes in natural and organic foods. Our intention had been to sample a set of individuals with much deeper experience with the category. Our screening questions indicated that these consumers purchased chicken roughly 15% more frequently than the grocery store shoppers (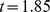; 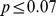). As the Big Carrot only carries organic products, these individuals also almost certainly had greater exposure to the category. Going forward, we refer to this subset of respondents as “enthusiasts” for the organic label. Table 1 provides some descriptive information about the respondents.

**Table 1:** Demographics of the sample.

	All	Enthusiasts
Gender		
Male	43%	18%
Female	57%	82%
		
Age		
18–34	55%	27%
35–50	26%	38%
51–65	15%	27%
Over 65	4%	8%
		
Education		
< High school	5%	4%
High school	31%	23%
College	51%	55%
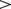 College	13%	18%
N	571	96

Each survey required roughly 15–20 minutes to complete. After reading an introductory text, respondents spent the majority of their time engaged in evaluating a set of forced comparisons in hypothetical choices, the choice task (described in detail below). Following the choice task, they answered questions relating to their beliefs about poultry and their concerns about health and food, as well as questions about their primary language, grocery shopping behavior, education and age. The research assistants also recorded the gender of each respondent.

Our approach offers something of a middle ground. Much of the psychological research on categories has been in the laboratory, evaluating either the effects of natural language categories, such as furniture, or of synthetic categories, such as patterns of dots. The sociological and managerial research on categories, meanwhile, has primarily used archival data to examine the effects of membership in categories on performance and other outcomes. Our current study has two chief advantages vis-à-vis these literatures. First, it examines a meaningful (and legitimated) category and therefore has greater external validity for the research on social categories than the extant psychological research. Second, with respect to the managerial and sociological literatures on categories, our design affords greater insight into how people actually perceive and value categories relative to attributes and therefore begins to open the black box of the socio-cognitive processes involved.

As with any research design, however, our approach also has its limitations. Given the complexity of completing the choice task, we decided that interviewers would need to guide respondents, which essentially precluded the selection of a random sample. Therefore, though the demographics of our respondents appear similar to the residents of the greater Toronto area, our results may not extrapolate to the population as a whole. Also, as discussed in the next section, the time required to complete the choice task limited the number of attributes that we could investigate.

### Measures

#### Choice task

To understand how respondents valued the organic category relative to a subset of attributes, much of the survey asked respondents to choose between sets of paired comparisons. The forced comparison of choices has been shown to have excellent within- and out-of-sample predictive power for actual choice [25], [26]. It therefore provides an excellent method for establishing the underlying valuations that audiences place on categories and attributes.

This technique involves creating a library of potential product profiles, choosing pairs of profiles out of this library and having respondents indicate whether they prefer one or the other or feel indifferent between them. In creating these profiles, we wanted them to seem as realistic as possible, incorporating the four dimensions of information that a shopper would normally see on a package: the brand, the category label, some features and the price.

We nevertheless faced two limitations in creating our sample profiles, both a function of combinatorics. To understand the problem, imagine that one wanted to test three brands, a category label, the presence or absence of three binary attributes and four price levels. Such a combination of characteristics would produce 192 potential profiles (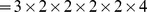), entailing more than 18,000 dyadic comparisons (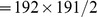).

One can reduce the number of comparisons that one must sample through the use of a fractional factorial design, which selects a subset of profiles in such a way as to ensure that each dimension remains orthogonal – and therefore uncorrelated – with every other dimension. This approach has the advantage of dramatically reducing the number of choices required to estimate unbiased values but the disadvantage of precluding the estimation of at least some of the interaction effects between characteristics. However, even the most efficient fractional factorial designs would still dictate well over one thousand comparisons in the 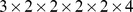 example above. We therefore had to limit strictly the number of dimensions and the number of levels on each dimension.

**Figure 1:**
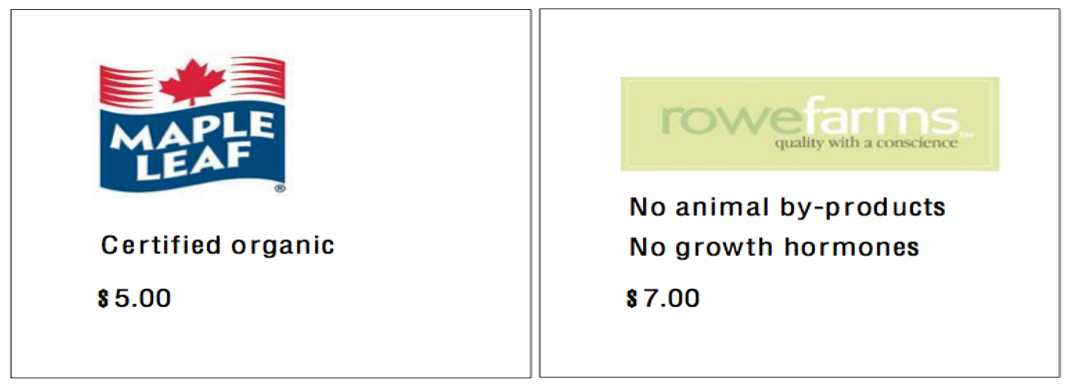
Two sample profile cards.

We created our profiles by combining levels from three dimensions (Figure 1 depicts two profile cards): (1) Category/attribute, (2) Brand, and (3) Price. Beginning with category/attribute, each profile included one of four possible indications: (i) grain fed, (ii) certified organic (depicted on the left panel), (iii) no animal byproducts, and (iv) no animal byproducts or growth hormones (depicted on the right panel). We chose “grain fed” as the baseline attribute profile because it conveyed no information: all poultry have grain as the primary component of their diets. “Organic” provided one level as we wanted to compare its value to that of attributes. Of the three attributes that distinguish organic poultry from conventional poultry, we included “no animal byproducts” over the other two because including “fed organic grain” as an attribute would have limited our ability to parse the value of the label from the attribute (since this attribute carries the organic label itself) and because our discussions with the CFIA made it clear that consumers cared more about animal byproducts than about the use of antibiotics. We included the “no animal byproducts or growth hormones” dual-attribute as our final level because we had an interest in whether a placebo attribute would influence valuations and because the CFIA indicated that growth hormones represented the second-most-commonly raised public concern (despite the fact that Canada had banned them in the 1970s). Many poultry producers in Canada also include the “no growth hormones” claim on their labels despite the fact that it does not differentiate them from other producers.

For realism, each profile also included a price – $5 or $7 (per kilogram) – and one of two possible brand names: Maple Leaf or Rowe Farms. Both represent actual brands. Maple Leaf supplies mainstream groceries and has a positioning akin to Oscar Meyer in the United States. It did not sell organic products at the time of the survey. Rowe Farms, meanwhile, occupies an upscale niche and emphasizes its quality and connection to smaller, local farmers. However, like Maple Leaf, it also did not offer organic products.

These three dimensions produce 16 potential profiles (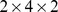). Although a complete comparison across all of these 16 profiles would have required 120 pairings, the use of a fractional factorial design allowed us to limit the choice task to 48 dyadic comparisons [27]. Even with this limited set, however, fatigue could still become a problem. To prevent this issue from influencing our results (and to eliminate other sorts of sequence effects), we rotated the order in which we presented the comparisons across the respondents. Specifically, we created a counter-balanced Latin square design, meaning that not only did the sequencing rotate but also each pairing followed every other pairing once and only once (across every group of 48 respondents).

#### Psychological state

We randomly assigned the negative affect condition to respondents and introduced it through the use of a prime. Before beginning the choice task, interviewers asked subjects to read and count the number of nouns in a short piece of text. Half of the respondents received the neutral prime, an account of the benefits of a heart-healthy diet (the “Mediterranean diet”). The other half of the respondents received a prime to place them in a negative mood. This text provided a factual account of the fatal, neurodegenerative disease in cattle known as bovine spongiform encephalopathy or, more colloquially, “mad-cow” disease.

For our purposes, spongiform encephalopathy has the useful property of being limited to ruminants. Poultry cannot contract it. The prime should therefore raise general concerns about health and safety but should not induce any particular preferences about poultry on the respondents. To test to see whether this manipulation worked, after the choice task, we asked respondents to indicate the degree to which they worried about (i) salmonella (a bacteria found in poultry that can cause food poisoning), (ii) their general health and (iii) the freshness of the food that they purchase (each on a four-level Likert scale). Consistent with our expectations, respondents exposed to the negative mood prime had higher levels of anxiety about their health (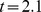, 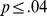) and about the freshness of food (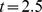, 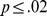) but not about salmonella (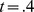, 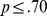).

### Estimation

The logic behind extracting valuations from the forced choices comes from considering them to be the results of linear, additive preference functions. Imagine that the utility (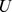) that individual, 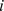, derives from a particular profile, 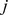, follows equation 1:
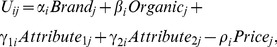
(1)


where 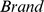 takes a value of 1 for Rowe Farm (versus 0 for the Maple Leaf baseline), 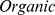 equals 1 if the profile includes the category label, 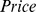 takes a value of 1 for the $ 5 condition, 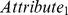 indicates the “no animal byproducts” attribute and 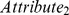 the “no animal byproducts and no hormones” dual attribute. The remaining terms – 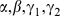 and 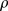 – meanwhile capture the valuations that the individual (implicitly) assigns to these characteristics.

One can then think about the probability of preferring one profile of a pair over the other, profile 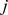 versus profile 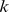, as being a matter of comparing the value associated with each and choosing the one that offers greater expected satisfaction:
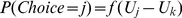
(2)


A number of techniques have been proposed for estimating the value coefficients, also known as partial utilities [26]. Market researchers, for example, frequently use linear optimization to derive them. Regression, however, has the benefit of allowing us to estimate standard errors.

Assuming that relative utility has a linear relationship to the probability of being chosen allowed us to estimate the coefficients with least squares regression (probit and logit regression yielded qualitatively equivalent results). In particular, we regressed a dependent variable equal to 1 if the respondent chose 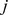, -1 if the respondent chose 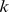 and 0 if the respondent felt indifferent between the two choices, and regressed it on the difference in the characteristics vectors for profiles 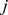 and 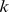. To accommodate potential correlations in errors within individuals across comparisons and within profiles across individuals, we report the Huber-White sandwich estimates of variance [28].

## Results

### Category meaning

Before turning to valuation of the category label relative to its requisite attributes, we first examined what respondents understood as the criteria for category membership. To do so, we coded the attributes mentioned in response to an open-ended question that simply asked them to describe the differences between organic and regular chicken. Table 2 reports all attributes named by at least five people (1% of the sample). The second column reports the proportion of all respondents mentioning an attribute, the third column the proportion mentioning it among enthusiasts (Big Carrot shoppers), and the final column the 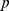-value for a 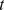-test of the difference between the proportions for enthusiasts and non-enthusiasts.

**Table 2:** Beliefs about the attributes of organic poultry.

Attribute	Full sample	Enthusiasts	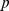 -value
No idea	24%	10%	0.00
			
Difference in feed	22%	16%	0.09
*Free range/cage free*	*21%*	*26%*	*0.19*
*No growth hormones*	*21%*	*23%*	*0.65*
Fed organic grain	11%	11%	0.93
*Fed grain*	*10%*	*7%*	*0.31*
Not fed animal byproducts	6%	7%	0.72
*No chemicals used*	*6%*	*4%*	*0.38*
No antibiotics	5%	10%	0.00
*Natural*	*5%*	*3%*	*0.38*
*Tastes better*	*4%*	*4%*	*0.86*
Certification	2%	5%	0.06
			
Correct definition	0%	0%	1.00
N	571	96	

We would begin by noting that nearly one-quarter of all respondents had no idea about the difference between organic and conventional chicken. Of those that mentioned an attribute, respondents most commonly thought that the two differed in their feed. That is correct but somewhat incomplete since these respondents did not understand the differences in the feed across the two categories. Being raised cage-free or outdoors came next in line. Interestingly, that feature does not actually differentiate organic poultry from conventional poultry in Canada. All birds are raised cage-free and none are raised outdoors (though all generally have access to the outdoors). The third-most-common belief held that organic poultry has not been fed growth hormones. As with being cage-free, while true, this characteristic also does not distinguish organic poultry from conventional poultry since Canada outlawed the use of growth hormones forty years ago. To highlight these inaccurate beliefs about the category definition, we have italicized them in the table. Note that more than half of the attributes mentioned do not distinguish organic poultry from non-organic poultry.

Much smaller proportions of the population named the actual requisite features: 11% recognized that organic poultry must receive organic feed; 6% understood that this feed could not contain animal byproducts; and 5% knew that the definition also meant that farmers could not use antibiotics in raising the animals. But not one respondent named this configuration of three distinguishing attributes as the definition, even if we generously allow the generic “difference in feed” to imply being fed organic grain.

One might nonetheless claim that this does not offer a fair test. After all, the organic category had been around for more than two decades before the Canadian government decided to codify its use as a label. Perhaps consumers had a clear understanding of the mapping of the category to attributes but the government simply did not follow it. In that case, however, even though the audience might not understand the legal definition, one would still expect a high degree of consensus in their beliefs about the attributes necessary for membership [10]. But our respondents did not even meet that lower bar. No more than one-quarter agreed on any one attribute and no more than five percent agreed on any particular combination of attributes.

Enthusiasts did not fair much better. Although only 10% of them did not mention an attribute, they actually held more inaccurate beliefs: 62% of the attributes that they mentioned – versus 51% for non-enthusiasts – do not actually distinguish organic poultry from conventional poultry (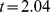; 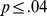). But they did have more consistent beliefs about these attributes: For each group – enthusiasts and non-enthusiasts – we calculated a Simpson index of attribute configurations, which essentially represents the probability that two individuals drawn at random would have the same set of beliefs [29]. It ranges from 0 to 1, with smaller values indicating greater diversity. We calculated a value of .104 for enthusiasts (more-experienced individuals) versus one of .068 for non-enthusiasts (less-experienced individuals). Individuals with greater experience with the category had more consistent beliefs about its requisite attributes (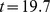; p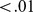). These individuals did not, however, associate the category label with a longer list of features as the feature creep conjecture would predict.

### Category valuation

To assess relative valuations, we analyzed the respondents' preferences for one profile over another. Table 3 reports the results of the initial choice regressions. Model 1 estimated the utility associated with each characteristic across all respondents in the neutral treatment. The “organic” category label, the “no animal byproducts or growth hormones” attribute set, the Rowe Farms brand name and lower price all had positive utility. Because our estimation used an additive, linear model and the dependent variable ranged from 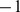 to 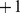 – a two-unit interval – one can directly interpret these values in terms of changes in the probability of choosing an option with a particular characteristic if one simply divides its coefficient by two. For example, the organic label increased the probability of an option being chosen by 14 percentage points (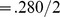) over a profile that did not contain the label. One could also scale the results by using the fact that a two-dollar change in price shifts the probability of preferring an option by 21 percentage points (dollar equivalents reported in Table 4). Hence, the average audience member attached a value of roughly $ 1.31 (per kg) to the organic label (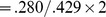).

**Table 3:** Estimated value of poultry characteristics.

	Neutral prime		Negative prime	
	All	Enthusiasts	All	Enthusiasts
	Model 1	Model 2	Model 3	Model 4
Organic	.280 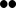	.693 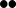	.280 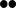	.693 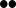
	(.017)	(.037)	(.017)	(.037)
No animal byproducts	−.027	.055	−.027	.055
	(.016)	(.036)	(.016)	(.036)
No byproducts or hormones	.250 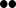	.471 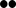	.250 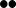	.471 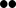
	(.016)	(.034)	(.016)	.034
Rowe Farms	.112 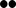	.351 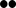	.112 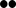	.351 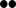
	(.011)	(.024)	(.011)	(.024)
Price	.429 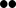	.278 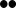	.429 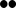	.278 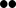
	(.011)	(.025)	(.011)	(.025)
Negative prime			.008	.008
			(.012)	(.028)
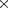 Organic			.042 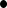	−.098 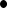
			(.024)	(.053)
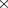 No anim byprod			.041 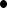	.014
			(.022)	(.050)
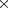 No byproducts			.081 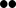	−.038
or hormones			(.022)	(.048)
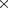 Rowe Farms			.037 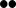	.092 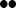
			(.015)	(.033)
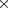 Price			−.056 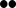	.019
			(.016)	(.036)
Constant	−.035 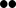	−.052 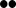	−.035 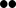	−.052 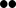
	(.008)	(.014)	(.007)	(.020)
R 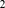	.12	.26	.12	.27
Observations	13,824	2,304	13,632	4,608
Individuals	288	48	571	96

Robust standard errors reported in parentheses; 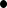 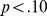, 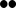 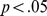.

**Table 4:** Valuations of characteristics (scaled in dollars).

	Neutral prime		Negative prime	
	All	Enthusiasts	All	Enthusiasts
	Model 1	Model 2	Model 3	Model 4
Organic	$1.31	$4.99	$1.73	$4.01
No animal byproducts	−$0.13	$0.40	$0.08	$0.46
No byproducts or hormones	$1.16	$3.39	$1.77	$2.92
Rowe Farms	$0.52	$2.53	$0.80	$2.98

In this baseline condition, respondents strongly preferred the category label, organic, over the primary differentiator, no animal byproducts (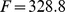, 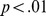). They also preferred the category over the “no animal byproducts or growth hormones” attribute combination, though by a much smaller margin (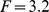, 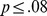). That fact surprised us. Recall that the Canadian government bans the use of growth hormones for all poultry. The “no animal byproducts or growth hormones” condition, therefore, does not differ meaningfully from the “no animal byproducts” condition. Yet the audience clearly perceived it as different. It's worth noting, moreover, that research has found that the mere perception of even non-existent ingredients can also influence the perceived taste of foods [30]. These inequalities held even more strongly among enthusiasts: They too valued the label significantly more than either “no animal byproducts” (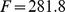, 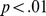) or “no animal byproducts or growth hormones” (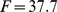, 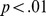) but exhibited the same surprising preference for the (non-informative) “no animal byproducts or growth hormones” condition over the “no animal byproducts” condition.

Model 2 demonstrates that enthusiasts, those experienced with members of the category, valued the category more highly than those less experienced with it. The organic label increased the probability that enthusiasts would choose an option by more than twice as much as non-enthusiasts: nearly 35 percentage points (equivalent to a roughly five-dollar change in price). One might worry, however, that this difference reflects selection rather than greater exposure to category members. Maybe enthusiasts simply value the same characteristics more highly. We address this issue below.

Models 3 and 4 next shift to the issue of whether the basis for evaluation changes depending on mood. We approached the analysis conservatively, allowing the mood manipulation to influence the valuation of any characteristic—that is, we estimated fully-interacted models. Because the “main” effects essentially capture the valuations in the neutral condition, they should be (and are) identical to the first two columns. For ease of interpretation, the associated columns in Table 4 combine the main and interaction effects. The interaction effects then capture how respondents' preferences differed as a function of their mood. On average, a negative mood increased the value associated with the organic label, the “no animal byproducts” attribute, the “no animal byproducts or growth hormones” combination and the Rowe Farms brand, and decreased the attractiveness of a low price.

To the extent that the negative mood manipulation shifts participants to paying attention to the details, one would expect that the attributes would increase in valuation relative to the category label. Although in the expected direction, the evidence here appears weak: The organic designation increased less in value than the “no animal byproducts or growth hormones” condition, though the difference reached only a marginal level of significance (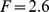, 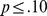). Among enthusiasts, exposure to the negative prime decreased the value of the organic label relative to that of the “no animal byproducts” condition (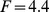, 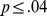) but not relative to the dual attributes (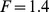, 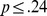).

However, as noted above, consumers did not appear aware of the attributes required for the organic designation. The mixed results therefore may reflect the fact that people had their own understandings of the meaning of the category label (i.e. the features that it represents). If respondents believed (even falsely) that the category encoded multiple attributes, their estimated valuations for the category may simply reflect their summed valuations of all of these attributes.

#### Audience-defined attributes

Although the constraints of designing a Latin square of tradeoffs did not permit an examination of the complete relationship between the category value and attributes directly, the open-ended question on the differences between organic and conventional poultry nevertheless allowed us to delve more deeply into these relationships. To address this issue, we re-estimated a series of models using respondents' reported beliefs about the distinguishing attributes (Table 2) in place of the organic label in the regression. In other words, if a respondent thought that “no growth hormones” differentiated organic from conventional fare, then we included an indicator variable – for beliefs about no growth hormones – to all of her product profiles that included the organic label, allowing us to estimate directly the value that respondents, on average, placed on these attributes. These models not only allow for the audience to have an understanding of the category that differs from its legal definition but also allow beliefs about these requisite attributes to vary across respondents.

In these models, the coefficient for the organic label captures the residual value attached to the label above and beyond the value of the attributes people believed that it represented. Since we could not associate the category with attributes for those who responded that they had no idea of what differentiated organic poultry from conventional poultry (“no idea” on Table 2), we excluded them from this estimation. Dropping them allowed us to distinguish the residual effect of the category above and beyond its perceived attributes from the value of the category for those respondents who simply did not have beliefs about its attributes. Due to collinearity in the vector of belief variables, we could not estimate a coefficient for the effect of believing that organic meant not being fed animal byproducts for enthusiasts in the neutral prime. Note also that because of the small number of individuals that named it as a belief, we could not include “certification” in the vector of attributes differentiating organic and conventional poultry.

Table 5 reports the results of these regressions. We believe that a few items in this table deserve attention. First, the inclusion of these beliefs generally improved the fit of the model. People varied in their beliefs about the meaning of the category and these beliefs in turn helped to explain some of the heterogeneity across individuals in the values that they accorded to the organic label. Second, the category label had a positive and significant residual value in all four groups. Even after accounting for what people believed to be the distinguishing attributes of the category, they still valued the category label itself. Third, enthusiasts valued the category more highly, even after controlling for their potentially stronger preferences for its underlying attributes. Fourth, note that the groups valued the “no animal byproducts or hormones” dual attribute more highly than they did the category when they believed the category meant no animal byproducts or growth hormones.

**Table 5:** Estimated value based on self-reported category meaning.

	Neutral prime		Negative prime	
	Non-enthusiasts	Enthusiasts	Non-enthusiasts	Enthusiasts
	Model 5	Model 6	Model 7	Model 8
Organic	.222 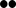	.641 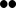	.431 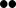	.586 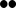
	(.046)	(.092)	(.042)	(.088)
Meaning of organic				
Difference in feed	.005	−.365 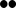	−.175 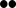	.148
	(.048)	(.102)	(.044)	(.103)
Free range/cage free	.003	.262 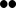	−.126 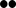	−.098
	(.042)	(.097)	(.043)	(.096)
No growth hormones	.053	.110	.009	−.064
	(.044)	(.119)	(.042)	(.109)
Fed organic grain	−.070	.279 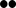	−.267 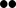	.271 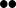
	(.054)	(.125)	(.055)	(.095)
Fed grain	−.148 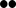	−.313 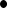	.008	−.079
	(.051)	(.176)	(.053)	(.112)
Not fed animal byproducts	.050		.010	.018
	(.075)		(.058)	(.087)
No chemicals used	.107	−.028	−.010	−1.30 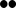
	(.074)	(.150)	(.058)	(.137)
No antibiotics	.114	.169	.158 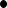	.489 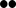
	(.080)	(.113)	(.083)	(.115)
Natural	.054	.029	.066	−.242
	(.088)	(.196)	(.063)	(.291)
Tastes better	−.046	.591 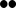	−.033	.257 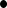
	(.102)	(.134)	(.080)	(.134)
No animal byproducts	−.022	.068 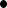	−.006	.090
	(.020)	(.037)	(.020)	(.036)
No byproducts or hormones	.275 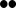	.504 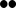	.332 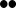	.455 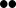
	(.020)	(.034)	(.020)	.035
Rowe Farms	.112 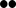	.409 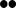	.137 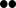	.446 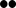
	(.014)	(.024)	(.014)	(.024)
Price	.460 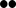	.284 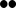	.371 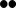	.248 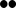
	(.014)	(.026)	(.014)	(.026)
Constant	−.039 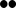	−.055 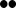	−.024 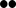	−.046 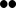
	(.011)	(.020)	(.011)	(.020)
R^2^	.14	.33	.12	.30
Observations	8,208	2,016	8,304	2,112
Individuals	171	42	173	44

Robust standard errors reported in parentheses; 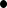 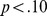, 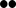 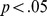.

These second, third and fourth findings suggest that audiences value the category label above and beyond the attributes required for membership in the category, perhaps due to feature creep, value creep or certification processes. To distinguish feature creep and certification from value creep, one can examine whether the value of the category label varied with the number of features that an individual associated with it. If the value of the category label arises from averaging the values accorded to accepted members of the category, then one would expect a relatively invariant relationship between the value of the label and its perceived attributes. But if the value of the label stems from being perceived as providing information about features not explicitly observed then its value should rise with the number of attributes the audience associates with it.

To investigate this issue, we used the responses to the open-ended question, reported in Table 2, to split individuals into three groups according to the number of features that they associated with the label: those who had no idea of the meaning (0 attributes), those who associated the category with one to three attributes, and those who associated it with four or more features. We then estimated the value of the organic label, as well as all other features, separately for each of these three groups. Although we estimated interactions between these groups and all of the attributes, Table 6 reports only the items of interest, the coefficients and standard errors for the organic label within each of these three groups. All three groups valued the category above and beyond its associated attributes, but the incremental value of the category label rose with the number of attributes that the individual associated with it, providing evidence in favor of feature creep and/or certification as central mechanisms underlying the extra value associated with category labels.

**Table 6:** Estimated value of category based on extensiveness of category beliefs.

	Organic	
	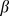	SE
No idea (0 attributes)	.139	.034
1–3 attributes	.321	.019
4+ attributes	.522	.132

## Discussion

Although the label “organic” has a clear definition for poultry in Canada, Canadian consumers exhibited little understanding of the distinguishing characteristics necessary to qualify for the label. In fact, many of the attributes they believed to be conditions for qualification are common to both organic and conventional poultry. Not only did the audiences not understand the “organic” definition, but they did not even display any substantial degree of consensus over its meaning. Enthusiasts – those with greater experience with the category – fared little better.

Despite their limited understanding of the meaning of the category, consumers nevertheless always preferred products with the “organic” label to those meeting its legal definition (but without the label), as well as to those with the attributes that they believed organics to have (but without the label). Those with more experience with the category and who associated more attributes with the category valued it even more highly.

These findings seem difficult to reconcile with the idea that categories serve as an efficient cognitive shorthand for sets of attributes [9], [31]. If categories simply mapped onto attribute sets, then one would expect audiences to understand their definitions and to value the label equivalently to the set of attributes that it represented. The findings do, however, fit with the expectations that one would have if categories represented an alternate means of organizing the world [32].

These results also seem surprising in light of recent evidence suggesting that audiences prefer crisp categories – those with clear boundaries – over more lenient ones – those with more ambiguous definitions [17], [33]. We would note, however, that these ideas about the advantage of crispness have been in terms of competition among categories. The organic label does not have any real competitors at the moment and therefore neither consumers nor producers can opt for a label with greater precision.

More broadly, category-level competition may have some interesting and somewhat counter-intuitive effects on the value of category labels. By selecting more lenient labels out of the system, competition may produce categories with more homogenous members, thereby producing categories less prone to feature creep, to value creep and to the need for certification. By doing so, category-level competition may interestingly limit the value of the label to being little more than the sum of the values associated with its underlying features.

Although our results provide evidence that the organic label has acquired value above and beyond that of its associated attributes, this finding represents but a single category. In thinking about how the premium accorded to the label might vary from one category to the next, we have two immediate expectations. First, we would expect a positive association between the value of the label and the complexity of the category. To the extent that audiences cannot enumerate the relevant attributes and would probably not even have the ability to examine them if they could, one would expect an even greater reliance on the category label in judgments of valuation.

Second, we would expect a positive association between the premium associated with a category label and its legitimacy for two reasons. On the one hand, the fact that a category label has been generally accepted may limit the perceived need for examining the meaning of and for understanding the label [34]. Audiences therefore might have a high degree of consensus on membership in the category while simultaneously not agreeing on the rules for membership. On the other hand, to the extent that audiences infer the value of the label from its associations, one might expect these associations to expand as audiences become more experienced with the category and consequently develop deeper associations between it and various attributes—ironically, from a dispersion of understandings about the category meaning rather than a sharpening of them.

To the extent that category labels do acquire a value of their own that potentially increases with legitimacy, it raises interesting issues for industry dynamics. Entrants – particularly those with novel products or organizational forms – face liabilities of newness due to the difficulty of developing reliable and accountable internal processes [35], [36]. If the category label has value above and beyond the attributes of the organizations, products and services being classified, it places entrants between a rock and a hard place. Failure to conform to the operations and offerings of incumbents – not just having all of the features of the incumbents but also, just as crucially, not having additional ones – may lead to entrants being perceived as less than full members of the category and therefore being viewed by consumers as offering less valuable goods and services. But entering with products identical to those already available dooms them to competing on price and quality against established players with greater scale and experience.

Given this bind, it's perhaps not surprising that entrepreneurial entry in many industries coincides with attempts to establish novel categories. When evaluated as a minicomputer, the machines that eventually become known as computer workstations could not compete on price or performance [18]. Software companies constantly struggle to define spaces that allow them to highlight their novel features without violating the expectations associated with a class of program [33]. When successful, these attempts, perhaps depending on the functional substitutability of the product being introduced, may either lead to the emergence of a new industry or the partitioning of an existing one [11].
