# Correction: Valuation, Categories and Attributes

**DOI:** 10.1371/journal.pone.0112038

**Published:** 2014-10-22

**Authors:** The PLOS ONE Staff

There are errors in the Author Contributions. The correct contributions are: Conceived and designed the experiments: IG. Performed the experiments: IG. Analyzed the data: IG OS. Wrote the paper: IG OS.
